# Evolution of Cryoglobulinemia in Direct-Acting Antiviral-Treated Asian Hepatitis C Patients With Sustained Virological Responses: A 4-Year Prospective Cohort Study

**DOI:** 10.3389/fimmu.2022.823160

**Published:** 2022-03-08

**Authors:** Ming-Ling Chang, Jur-Shan Cheng, Ya-Hui Chuang, Li-Heng Pao, Ting-Shu Wu, Shiang-Chi Chen, Ming-Yu Chang, Rong-Nan Chien

**Affiliations:** ^1^ Division of Hepatology, Department of Gastroenterology and Hepatology, Chang Gung Memorial Hospital, Taoyuan, Taiwan; ^2^ Department of Medicine, College of Medicine, Chang Gung University, Taoyuan, Taiwan; ^3^ Clinical Informatics and Medical Statistics Research Center, College of Medicine, Chang Gung University, Taoyuan, Taiwan; ^4^ Department of Emergency Medicine, Chang Gung Memorial Hospital, Keelung, Taiwan; ^5^ Department of Dermatology, Chang Gung Memorial Hospital, Taipei and Linkou, Taiwan; ^6^ Graduate Institute of Health-Industry Technology, Chang Gung University of Science and Technology, Taoyuan, Taiwan; ^7^ Research Center for Industry of Human Ecology, Chang Gung University of Science and Technology, Taoyuan, Taiwan; ^8^ Department of Dermatology, Chang Gung Memorial Hospital, Taipei, Taiwan; ^9^ Department of Nursing, Taipei Medical University, Taipei, Taiwan; ^10^ Division of Pediatrics, Chang Gung Memorial Hospital, Keelung, Taiwan

**Keywords:** cryoglobulinemia, DAA, HCV, SVR, cryoglobulinemic vasculitis

## Abstract

**Background:**

How cryoglobulinemia evolves after sustained virological response (SVR) following direct-acting antiviral (DAA) treatment in Asian hepatitis C virus (HCV)-infected patients remains elusive.

**Methods:**

A prospective cohort study was conducted in 422 Taiwanese patients (358 completed DAA therapy and 353 experienced SVRs). Serum cryoglobulins were surveyed at baseline and every 3-6 months posttherapy.

**Results:**

Of 422, 227 (53.8%) had cryoglobulinemia, 8 (1.89%) had cryoglobulinemic vasculitis. Of 227, 54 (23.8%), 57 (25.1%) and 116 (51.1%) had 1, 2 and 3 cryoglobulins, respectively; those with 3 cryoglobulins had the highest alanine aminotransferase, immunoglobulin G (IgG) and fibrosis-4 index. During a 4-year follow-up, among SVR patients, cryoglobulinemia rates decreased from 56.4% to 15.4%, single cryoglobulin rates increased (21.6% to 63.9%) and 3 cryoglobulin rates decreased (55.7% to 11.1%). Compared with baseline values, among SVR patients with baseline cryoglobulinemia, complement component 4 levels increased, and IgG and IgM levels decreased until 48 weeks posttherapy for those without posttherapy cryoglobulinemia. All 8 cryoglobulinemic vasculitis patients exhibited SVRs; 5 (62.5%) achieved complete clinical response 12 weeks posttherapy, of whom, 2 (40%) experienced clinical relapse 24~48 weeks posttherapy. Baseline IgM levels were associated with posttherapy cryoglobulinemia in SVR patients (cut-off values at 12, 24, 48 weeks and 4 years posttherapy: 130, 105, 118 and 168 mg/dL, respectively).

**Conclusions:**

Among DAA-treated SVR patients, in 4 years, cryoglobulinemia rates decreased from 56.4% to 15.4%, multiple cryoglobulin rates decreased, cryoglobulinemia signals reversed, 62.5% of cryoglobulinemic vasculitis patients achieved complete clinical response (40% had relapse), and baseline IgM levels indicated posttherapy cryoglobulinemia.

## Introduction

Hepatitis C virus (HCV) is a human pathogen responsible for acute and chronic liver disease. It is chronically infecting an estimated 71.1 million individuals worldwide (1) and classified into 8 genotypes (2). In addition to hepatic complications such as cirrhosis and hepatocellular carcinoma, HCV causes many extrahepatic complications, including mixed cryoglobulinemia (3), obesity, diabetes and dyslipidemia (4). Mixed cryoglobulinemia is the most common HCV-associated extrahepatic complication; up to 90% of patients with mixed cryoglobulinemia harbor HCV infection, and over 60% of patients with chronic HCV infection (CHC) have circulating mixed cryoglobulins (3, 5). Most of the information regarding HCV-associated mixed cryoglobulinemia came from studies conducted in Europe (6–8) or the USA (9). By contrast, in Asia, mixed cryoglobulinemia is an elusive condition. We previously showed that over 60% of Taiwanese patients with CHC had mixed cryoglobulinemia (3), and even among patients with spontaneous HCV clearance, approximately 1/3 had mixed cryoglobulinemia (10). Mixed cryoglobulinemia thus is not a minor issue for Asian individuals with HCV infection. Up to 8 years posttherapy, the prevalence of mixed cryoglobulinemia among CHC patients who achieved sustained virological responses (SVRs) following interferon-based anti-HCV therapy was still 17.64% (3). As interferon has immune-modulating functions, the persistence of mixed cryoglobulinemia after SVR may be induced by the withdrawal of interferon-based therapy (11). With the advent of direct-acting antivirals (DAAs), which target specific HCV proteins during its life cycle (12), anti-HCV treatment has resulted in a high cure rate with a short treatment duration in CHC patients, and the recovery pattern of HCV-associated mixed cryoglobulinemia may not be masked by immune-modulating effects. Several studies in Europe (13–17), Canada (18) and the USA (19) have shown the beneficial effects of DAA in treating HCV-associated mixed cryoglobulinemia, with complete clinical response rates ranging from 61 to 100%. Although some sporadic cases were reported in Japan (20–22), the precise effect of DAA in treating mixed cryoglobulinemia has mostly remained unidentified among CHC patients in Asia.

Accordingly, we sought to elucidate this effect in Taiwan, an Asian country where HCV infection is rampant (23), by conducting a 4-year prospective study analyzing serum cryoglobulins of CHC patients before and every 12-24 weeks after the completion of DAA therapy.

## Methods/Materials

### Patients

The study included subjects aged 18 years or older with CHC who had been surveyed for baseline cryoglobulinemia. Subjects with human immunodeficiency virus or hepatitis B virus infection, hemochromatosis, primary biliary cholangitis, primary sclerosing cholangitis, autoimmune hepatitis, immunosuppressive therapy for any reason, alcoholic liver disease or malignancy and recipients of solid organ transplants were excluded. CHC was defined as detectable HCV RNA for >24 weeks. Cryoglobulinemia was defined as positive for serum cryoglobulin, and cryoglobulinemic vasculitis was defined as described previously (3, 19).

### Study Design

A total of 422 CHC patients who had been surveyed for serum cryoglobulins were consecutively recruited at a tertiary referral center between May 2015 and December 2019. Of the 422 patients, 358 completed a course of anti-HCV therapy with various DAA combinations (**Supplementary Table 1**) according to the reimbursement policy of the Bureau of National Health Insurance of the country and were followed for ≥ 12 weeks after completion of DAA therapy. Several baseline factors, including sex, age, body mass index (BMI), HCV RNA, HCV genotype, single-nucleotide polymorphisms of interferon-λ3-rs12979860, cryoglobulin, estimated glomerular filtration rate (eGFR), total cholesterol (TC), triglycerides (TG), homeostatic model assessment for insulin resistance *(HOMA-IR)* [fasting insulin (μU/mL) × fasting glucose (mmol/L)/22.5], alanine aminotransferase (ALT), rheumatoid factor (RF), immunoglobulin G (IgG), IgM, complement component 3 (C3), C4 and fibrosis-4 (FIB-4) index [(age (years) × aspartate transaminase (U/L)/(platelets (10^9^/L) × (√(ALT (U/L))], and the presence of hepatic cirrhosis were surveyed and recorded for enrolled patients. Biochemical tests were performed at the clinical pathology laboratories of the hospital using routine automated techniques, serum cryoglobulins were measured using the double immunodiffusion method (24), hepatic cirrhosis was screened by abdominal sonography and confirmed by FibroScan. An SVR was defined as undetectable levels of HCV RNA 12 weeks after the completion of therapy. The clinical response of cryoglobulinemic vasculitis following SVR was considered complete if a patient’s Birmingham vasculitis activity score (version 3) was 0 or if all affected organs improved 12 weeks after the end of therapy (17). The cryoglobulins and the aforementioned variables were surveyed among the SVR patients every 12-24 weeks after the completion of therapy.

### Statistics

All statistical analyses were performed using the Statistical Package for Social Science (SPSS package version 21, SPSS Inc., Chicago, IL, USA) or MedCalc (MedCalc ver. 12.4, MedCalc Software Corp., Acacialaan, Ostend, Belgium) software. The continuous variables are summarized as the means +/- standard deviations (SDs), and the categorical variables are summarized as frequencies and percentages. Data were analyzed by one-way analysis of variance (ANOVA) when comparing variables from multiple groups. *Post hoc* analyses were performed with the least significant difference multiple comparison test. Multivariate logistic regression models were used to assess the relationships between various dependent and independent factors by adjusting for all independent variables with *p* values <0.1 in the univariate analyses. Hosmer-Lemeshow tests were performed to survey the goodness of fit for the multivariate logistic regression models. Receiver operating characteristic curve (ROC) analyses were performed to evaluate whether independent variables were significant predictors of the dependent variables. The Youden index was assessed to identify the optimal cut-off values of the independent variables according to the coordinate points of the ROC curves. Longitudinal alterations in profiles within the same SVR patients were analyzed and compared using ANOVA, employing general linear model-repeated measures. Paired t tests or nonparametric tests (Wilcoxon test) were performed for the same variable measured in the same subjects at >2 time periods when Mauchly’s sphericity tests yielded *p* values <0.05 during repeated measures. The 95% confidence intervals for mixed cryoglobulinemia rates were calculated based on the Poisson distribution. Statistical significance was defined at the 5% level based on two-tailed tests.

### Institutional Review Board

Written informed consent was obtained from each patient. The study protocol conformed to the ethical guidelines of the 1975 Declaration of Helsinki and was approved by the local institutional review board.

## Results

### Baseline Characteristics

Of 422 CHC patients, 227 (53.8%) had cryoglobulinemia, 8 (1.89%) had cryoglobulinemic vasculitis, and 65 had undergone previous unsuccessful interferon-based therapy. Of 65, 30 (46.5%) had cryoglobulinemia. Compared with patients without cryoglobulinemia, the patients with baseline cryoglobulinemia were more frequently female; had higher FIB-4 indexes, IgG and IgM levels; had higher cirrhosis rates; and had lower HCV RNA, TC, eGFR, C3 and C4 levels and platelet counts (**Table 1**).

**Table 1:** Baseline characteristics of patients with CHC.

	Total (n = 422)	Cryoglobulinemia (+) (n = 227)	Cryoglobulinemia (-) (n = 195)	*p* values
Female, n (%)	232 (54.3)	139 (61.2)	93 (47.7)	0.008
Age (years)	60.3+/-12.8	61.4+/-12.6	59.1+/-12.9	0.072
BMI (kg/m^2^)	24.6+/-3.8	24.3+/-3.8	24.9+/-3.9	0.07
Log HCV RNA (logIU/mL)	5.98+/-0.87	5.80/-0.87	6.14+/-0.83	<0.001
HCV genotype				
Genotype 1, n (%)	254 (60.2)	136 (59.9)	118 (60.5)	0.397
Genotype 2, n (%)	125 (29.6)	70 (30.8)	55 (28.1)	0.377
Genotype 3, n (%)	6 (1.4)	5 (2.2)	1 (0.5)	0.155
Others, n (%)	37 (8.8)	16 (7.0)	21 (10.7)	0.157
ALT(U/L)	79.7+/-90.3	76.6+/-56.4	83.9+/-118.6	0.4
FIB-4	3.52+/-3.38	4.29 +/-3.98	2.65+/-2.29	<0.001
Platelet (10^3^/mm)	177.0+/-71.7	165.3+/-75.6	190.6+/-68.8	0.003
Liver cirrhosis, n (%)	92 (21.5)	67 (29.5)	25 (16.7)	0.002
eGFR (ml/min/1.73m^2^)	86.7+/-41.9	87.9+/-41.9	91.5+/-40.5	0.019
Total cholesterol (mg/dL)	169.6+/-35.2	163.6+/-35.3	176.1+/-33.6	<0.001
Triglycerides (mg/dL)	102.8+/-52.5	100.2+/-50.9	103.5+/-46.3	0.495
HOMA-IR	3.17+/-4.88	2.90+/-2.96	3.34+/-6.30	0.353
C3 (mg/dL)	100.0+/-20.2	95.8+/-19.3	104.4+/-20.2	<0.001
C4 (mg/dL)	19.4+/-8.5	17.6+/-9.0	21.3+/-7.4	<0.001
IgG (mg/dL)	1802+/-478	1916+/-483	1675+/-442	<0.001
IgM (mg/dL)	114.5+/-60.4	136.4+/-66.2	90.5+/-42.4	<0.001
RF(IU/mL)	20.26+/-46.57	24.9+/-61.2	15.2+/-20.6	0.064

CHC, chronic hepatitis C virus infection; BMI, body mass index; ALT, alanine transaminase; FIB-4, Fibrosis-4; eGFR, estimated glomerular filtration rate; HOMA-IR, homeostasis model assessment-insulin resistance; C3, complement component 3; C4, complement component 4; IgG, Immunoglobulin G; IgM, Immunoglobulin M; RF, rheumatoid factor.

The cryoglobulin components of the 227 subjects with cryoglobulinemia are listed in **Table 2**. Of 227 patients, 54 (23.8%) had 1 cryoglobulin (with either IgM or IgG), 57 (25.1%) had 2 cryoglobulins (most were IgG and IgM), and 116 (51.1%) had 3 cryoglobulins (IgG, IgA and IgM). Among the 277 patients, those with 3 cryoglobulins had the highest ALT and IgG levels and FIB-4 indexes and the lowest platelet counts. Compared with those without cryoglobulinemia, those with 1 cryoglobulin had lower BMI, ALT and eGFR and higher IgM levels. Those with 3 cryoglobulins were more frequently female; were older; had higher FIB-4, IgG and IgM levels; and had lower HCV RNA, TC, TG, C3, and C4 levels and platelet counts (**Supplementary Table 2**).

**Table 2:** Baseline characteristics of 227 cryoglobulin-posive CHC patients.

	Single cryoglobulin [C1, n = 54 (23.7%)]	2 cryoglobulins [C2, n = 57 (25.1%)]	3 Cryoglobulins [C3, n = 116 (51.1%)]	*ANOVA p* values	*Post Hoc p* values
Female, n (%)	29 (53.7)	36 (63.2)	71 (61.2)	0.553	
Age (years)	59.8+/-12.85	59.7+/-12.2	62.9+/-12.7	0.185	
BMI (kg/m^2^)	23.44+/-3.37	24.8+/-3.89	24.4+/-3.87	0.145	
Log HCV RNA (logIU/mL)	6.03+/-0.85	5.79+/-1.00	5.76+/-0.81	0.149	
HCV genotype					
Genotype 1, n (%)	30 (55.6)	33 (57.9)	70 (60.3)	0.843	
Genotype 2, n (%)	18 (33.3)	19 (33.3)	33 (28.4)	0.680	
Genotype 3, n (%)	1 (1.9)	0 (0)	4 (3.4)	0.35	
Others, n (%)	5 (9.2)	5 (8.7)	9 (7.7)	0.877	
Cryoglobulin components					
IgG 1+, n (%)	18 (33.3)				
IgG 2+, n (%)	12 (22.2)				
IgM 1+, n (%)	24 (46.3)				
IgG 1+, IgA 1+, n (%)		1 (1.8)			
IgG 1+, IgM 1+, n (%)		21 (36.8)			
IgA 1+, IgM 1+, n (%)		1 (1.8)			
IgG2+, IgM 1+, n (%)		13 (22.8)			
IgA 2+, IgM 2+, n (%)		1 (1.8)			
IgG2+, IgM 2+, n (%)		20 (30.5)			
IgG1+, IgA1+, IgM 1+, n (%)			25 (21.6)		
IgG1+, IgA1+, IgM2+, n (%)			2 (1.7)		
IgG2+, IgA1+, IgM1+, n (%)			33 (28.4)		
IgG2+, IgA1+, IgM2+, n (%)			39 (33.6)		
IgG2+, IgA2+, IgM1+, n (%)			1 (0.9)		
IgG2+, IgA2+, IgM2+, n (%)			16 (13.8)		
ALT(U/L)	53.8+/-39.2	74.0+/-54.8	88.5+/-60.7	0.001	C1 vs. C3, * p< *0.001 C2 vs. C3, *p *= 0.048
FIB-4	2.74 +/-2.41	3.09+/-3.28	5.56+/-4.42	<0.001	C1 vs. C3, *p *< 0.001 C2 vs C3, *p *< 0.001
Platelet (10^3^/mm)	191.9+/-73.9	184.4+/-67.3	143.6+/-68.1	<0.001	C1 vs. C3, *p *< 0.001 C2 vs C3, *p *< 0.001
Liver cirrhosis, n (%)	8 (14.8)	13 (22.8)	40 (34.5)	0.018	C1 vs. C3, *p *= 0.006
eGFR (ml/min/1.73m^2^)	75.7+/-43.7	82.8+/-42.3	84.3+/-40.9	0.453	
Total cholesterol (mg/dL)	168.2+/-41.0	162.6+/-27.7	162.0+/-36.0	0.561	
Triglycerides (mg/dL)	116.9+/-70.6	101.1+/-48.7	92.2+/-38.7	0.014	C1 vs. C3, *p *= 0.004
HOMA-IR	2.67+/-3.74	2.76 +/-1.88	3.06+/-3.01	0.683	
C3 (mg/dL)	97.8+/-15.5	103.6+/-23.2	93.0+/-18.6	0.033	C2 vs. C3, *p *= 0.001
C4 (mg/dL)	18.9+/-7.5	20.1+/-14.2	16.5+/-7.3	0.123	
IgG (mg/dL)	1696.5+/-388	1798+/-418	2017+/-500	0.002	C1 vs. C3, *p *= 0.01 C2 vs. C3, *p *= 0.034
IgM (mg/dL)	134.0+/-62.2	108.8+/-46.5	145.1+/-70.4	0.036	C2 vs. C3, *p *= 0.01
RF (IU/mL)	14.03+/-7.45	16.5+/-19.1	30.6+/-75.9	0.322	

CHC, chronic hepatitis C virus infection; ANOVA, Analysis of variance; BMI, body mass index; IgG, Immunoglobulin G; IgA, Immunoglobulin A; IgM, Immunoglobulin M; ALT, alanine transaminase; FIB-4, Fibrosis-4; eGFR, estimated glomerular filtration rate; HOMA-IR, homeostasis model assessment-insulin resistance; C3, complement component 3; C4, complement component 4; IgG, Immunoglobulin G; IgM, Immunoglobulin M; RF, rheumatoid factor.

### Longitudinal Follow-Up of Cryoglobulinemia Among SVR Patients

**Figure 1:**
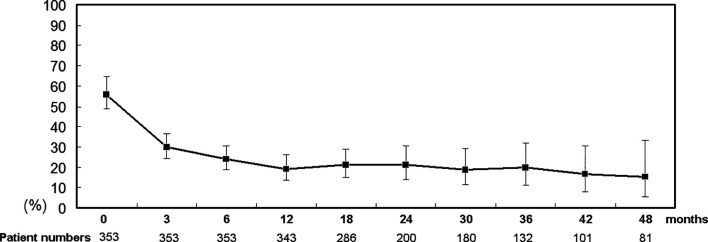
Percentage (95% confidence interval) of SVR patients with cryoglobulinemia. Time points: 0: baseline; 3, 6, 12, 18, 24, 30, 36, 42 and 48: 3, 6, 12, 18, 24, 30, 36, 42 and 48 months after completion of DAA therapy, respectively.

Of 358 patients who completed a course of DAA therapy, 65 (18.1%) had undergone previous unsuccessful interferon-based therapy, and 353 (98.6%) had achieved SVRs. During a 4-year follow-up (mean +/- standard deviation: 607+/-114 days, median: 567 days, range: 434 to 1460 days), among the 353 SVR patients, the rates of cryoglobulinemia decreased over time: from 56.4% at baseline to 15.4% at 4 years posttherapy (**Figure 1**); of the SVR patients with cryoglobulinemia, the rates of 1 cryoglobulin increased (21.6% to 63.9%) and that of 3 cryoglobulins decreased over time (55.7% to 11.1%). Although cryoglobulinemia rates decreased over time, the serum cryoglobulins in SVR patients varied during the follow-up period. The cryoglobulinemia rates among the SVR patients with and without baseline cryoglobulinemia were 27.6% and 9.0% at 4 years posttherapy, respectively (**Supplementary Table 3**). The most common combinations of immunoglobulins in the cryoglobulins were as follows: at baseline, IgG2+, IgA1+ and IgM2+ (10.2%); at 12 weeks posttherapy, IgM1+ (7.6%); at 24 weeks posttherapy, IgM1+ (4.2%) or IgG1+ (4.2%); at 48 weeks posttherapy, IgM1+ (3.4%); and at the final follow-up, IgG1+ (1.4%).

Among the SVR patients with baseline cryoglobulinemia, compared with baseline levels, at 12 weeks posttherapy, C3 and C4 levels increased and IgG, IgM and RF levels decreased for those without 12-week posttherapy cryoglobulinemia; at 24 and 48 weeks posttherapy, C4 levels increased and IgG and IgM levels decreased for those without cryoglobulinemia until 48 weeks posttherapy (**Supplementary Table 4**).

**Figure 2:**
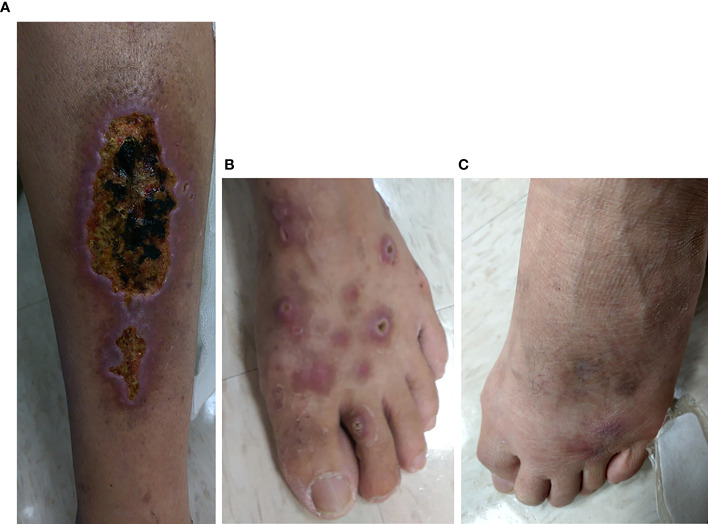
Leg lesions at baseline **(A, B)** and 3 years posttherapy **(C)** in a representative CHC patient with cryoglobulinemic vasculitis who achieved an SVR and a complete clinical response at 12 weeks posttherapy. **(A)** Multiple well-demarcated ulcers with black base and slightly elevated erythematous margins over the left calf of the patient. **(B)** Many punched-out, deep ulcers with crusting and elevated inflamed edges over the left dorsal foot of the patient. **(C)** Marked improvement in previous ulcers of the left dorsal foot of the same patient. However, newly developed, swollen, erythematous to purpuric papular lesions were noted at the roots of the third and fourth toes.

Of the 8 patients with baseline cryoglobulinemic vasculitis, 8 (100%) had SVRs, and 5 (62.5%) achieved complete clinical responses at 12 weeks posttherapy. However, 2 (40%) female patients of the 5 complete clinical responders experienced relapses. One experienced relapse at 24 weeks posttherapy, and then her symptoms and serum cryoglobulin disappeared; the other experienced relapses at 36 and 48 weeks posttherapy, and her serum cryoglobulins varied at 4 years posttherapy (undetectable at 12 and 24 weeks posttherapy, detectable at 48 and 72 weeks posttherapy, undetectable at 96 weeks posttherapy, and detectable after 144 weeks posttherapy). All relapses were associated with milder symptoms than those at baseline (**Figure 2**).

### Factors Associated With Posttherapy Cryoglobulinemia in SVR Patients

**Figure 3:**
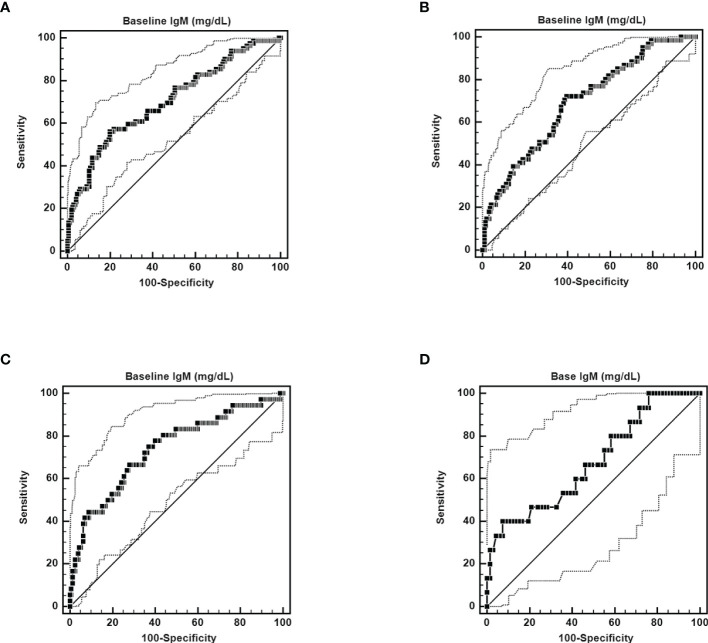
ROC curves of the ability of pretherapy IgM to predict 12-week posttherapy (cut-off value >130 mg/dL, AUC): 0.703, 95% CI AUC: 0.646-0.757, sensitivity: 56.10%, specificity: 80.10%, *p* < 0.0001) **(A)**, 24-week posttherapy (cut-off value >105 mg/dL, AUC: 0.691, 95% CI AUC: 0.630-0.747, sensitivity: 71.13%, specificity: 60.31%, *p* < 0.0001) **(B)**, 48-week posttherapy [cut-off value >118 mg/dL, AUC: 0.738, 95% CI AUC: 0.670-0.799, sensitivity: 66.67%, specificity: 71.97%, *p* < 0.0001) **(C)**, and 4-year posttherapy cryoglobulinemia (cut-off value >168 mg/dL, AUC: 0.677, 95% CI AUC: 0.564-0.776, sensitivity: 40.00%, specificity: 92.54%, *p* = 0.0287) **(D)**.

n SVR patients, baseline IgM levels were independently associated with 12-week and 24-week posttherapy cryoglobulinemia (**Supplementary Tables 5**, **6**), baseline BMI and IgM levels were independently associated with 48-week posttherapy cryoglobulinemia (**Table 3**), and baseline TC and IgM levels were independently associated with 4-year posttherapy cryoglobulinemia (**Table 4**). Baseline IgM significantly predicted posttherapy cryoglobulinemia, and the associated areas under the curve (AUCs) were as follows: 12-week posttherapy cryoglobulinemia, 0.703 (cut-off value: >130 mg/dL, *p*<0.001, **Figure 3A**); 24-week posttherapy cryoglobulinemia, 0.691 (cut-off value: >105 mg/dL, *p*<0.001, **Figure 3B**); 48-week posttherapy cryoglobulinemia, 0.738 (cut-off value: >118 mg/dL, *p*<0.001, **Figure 3C**); and 4-year posttherapy cryoglobulinemia, 0.677 (cut-off value: >168 mg/dL, *p*=0.033, **Figure 3D**).

**Table 3:** Univariate and multivariate analyses of baseline factors for 48-week post-therapy cryoglobulinemia in SVR patients.

	Univariate		Multivariate	
	OR (95% CI OR)	*p* values	OR (95% CI OR)	*p* values
Male, yes	0.398 (0.183-0.863)	0.02	0.514 (0.211-1.254)	0.144
Age (years)	1.025 (0.995-1.056)	0.097	1.015 (0.977-1.054)	0.449
BMI (kg/m^2^)	1.113 (1.021-1.212)	0.015	1.120 (1.011-1.241)	0.03
ALT(U/L)	0.995 (0.988-1.002)	0.157		
FIB-4	1.110 (1.016-1.212)	0.02	0.953 (0.801-1.134)	0.589
Platelet (10^3^/mm)	0.994 (0.989-1.000)	0.039	0.997 (0.989-1.006)	0.548
Liver cirrhosis, n (%)	3.144 (1.479-6.686)	0.003	1.516 (0.578-3.975)	0.397
eGFR (ml/min/1.73m^2^)	0.998 (0.988-1.007)	0.613		
Total cholesterol (mg/dL)	0.995 (0.984-1.007)	0.436		
Triglycerides (mg/dL)	0.997 (0.989-1.006)	0.533		
HOMA-IR	0.977 (0.879-1.085)	0.662		
C3 (mg/dL)	0.987 (0.969-1.005)	0.164		
C4 (mg/dL)	0.967 (0.921-1.026)	0.180		
IgG (mg/dL)	1.001 (1.000-1.002)	0.01	1.001 (0.999-1.002)	0.259
IgM (mg/dL)	1.016 (1.009-1.023)	<0.001	1.015 (1.008-1.023)	<0.001
RF(IU/mL)	0.997 (0.983-1.012)	0.69		

SVR, sustained virological response; OR, Odds ratio; CI, confidence invertal; BMI, body mass index; ALT, alanine transaminase; FIB-4, Fibrosis-4; eGFR, estimated glomerular filtration rate; HOMA-IR, homeostasis model assessment-insulin resistance; C3, complement component 3; C4, complement component 4; IgG, Immunoglobulin G; IgM, Immunoglobulin M; RF, rheumatoid factor.

**Table 4:** Univariate and multivariate analyses of baseline factors for 4-year post-therapy cryoglobulinemia in SVR patients.

	Univariate		Multivariate	
	HR (95% CI HR)	*p* values	HR (95% CI HR)	*p* values
Male, yes	0.461 (0.147-1.449)	0.185		
Age (years)	1.039 (0.988-1.094)	0.139		
BMI (kg/m^2^)	0.907 (0.765-1.765)	0.26		
ALT (U/L)	1.002 (0.993-1.011)	0.717		
FIB-4	1.088 (0.972-1.217)	0.142		
Platelet (10^3^/mm)	0.995 (0.986-1.003)	0.232		
Liver cirrhosis, n (%)	1.526 (0.511-4.554)	0.448		
eGFR (ml/min/1.73m^2^)	1.001 (0.984-1.018)	0.935		
Total cholesterol (mg/dL)	0.98 (0.959-1.001)	0.063	0.971 (0.947-0.995)	0.017
Triglycerides (mg/dL)	0.998 (0.987-1.008)	0.697		
HOMA-IR	0.828 (0.581-1.178)	0.293		
C3 (mg/dL)	0.985 (0.963-1.008)	0.197		
C4 (mg/dL)	0.942 (0.862-1.030)	0.19		
IgG (mg/dL)	0.999 (0.998-1.000_	0.251		
IgM (mg/dL)	1.007 (1.001-1.013)	0.032	1.010 (1.003-1.008)	0.008
RF(IU/mL)	1.006 (0.977-1.037)	0.683		

SVR, sustained virological response; HR, hazard ratio; CI, confidence invertal; BMI, body mass index; ALT, alanine transaminase; FIB-4, Fibrosis-4; eGFR, estimated glomerular filtration rate; HOMA-IR, homeostasis model assessment-insulin resistance; C3, complement component 3; C4, complement component 4; IgG, Immunoglobulin G; IgM, Immunoglobulin M; RF, rheumatoid factor.

## Discussion

The most compelling results of the current study were as follows:

(1) Of 422 CHC patients, 227 (53.2%) had baseline cryoglobulinemia; these patients were more frequently female; had higher FIB-4, IgG and IgM levels and cirrhosis rates; and had lower HCV RNA, TC, eGFR, C3 and C4 levels and platelet counts than patients without cryoglobulinemia. (2) Of 227 patients with baseline cryoglobulinemia, 54 (23.8%), 57 (25.1%) and 116 (51.1%) had 1 cryoglobulin, 2 and 3 cryoglobulins, respectively. Among patients with baseline cryoglobulinemia, those with 3 cryoglobulins had the highest ALT and IgG levels and FIB-4 indexes and the lowest platelet counts. (3) During a follow-up to 4 years, among the SVR patients, the cryoglobulinemia rates decreased over time (56.4% to 15.4%); among those with cryoglobulinemia, the 1 cryoglobulin rates increased and 3 cryoglobulin rates decreased over time. Among 8 patients with cryoglobulinemic vasculitis, 8 (100%) had SVRs, and 5 (62.5%) had complete clinical responses at 12 weeks posttherapy, but 2 of 5 (40%) complete clinical responders experienced relapses in 4 years. (4) Among the SVR patients with baseline cryoglobulinemia, compared with baseline levels, up to 48 weeks posttherapy, C4 levels increased and IgG and IgM levels decreased among those without any posttherapy cryoglobulinemia. (5) Baseline IgM significantly predicted posttherapy cryoglobulinemia in SVR patients.

Our previous study showed that the baseline rate of cryoglobulinemia in CHC patients was 64.5% (3), which was higher than that (53.2%) in the current study, in which 65 patients had undergone unsuccessful interferon-based therapy, and only 46.5% of the 65 patients had baseline cryoglobulinemia. This finding is consistent with the fact that mixed cryoglobulinemia was linked with a favorable interferon therapeutic response (25), and a lower cryoglobulinemia rate in patients with previous interferon treatment failure (46.5 vs. 64.5%) might reduce the average cryoglobulinemia rate in the current cohort. Compared with patients without cryoglobulinemia, patients with cryoglobulinemia had higher cirrhosis rates and FIB-4 indexes and lower TC levels and platelet counts, indicating that mixed cryoglobulinemia was associated with cirrhosis and poor cholesterol synthesis (3, 10), while patients with cryoglobulinemia had higher levels of IgG and IgM and lower levels of C3, C4 and HCV RNA, reflecting the paramount phenotype of mixed cryoglobulinemia (3, 10, 26). Thus, the baseline comparisons strengthen the reliability of our data.

Interestingly, approximately 1/4 (23.8%) and 1/2 (51.1%) of the patients with cryoglobulinemia had 1 cryoglobulin and 3 cryoglobulins, respectively. The term cryoglobulinemia refers to the presence of one (type I) or more immunoglobulins (types II and III) in the serum (27). Specifically, type I cryoglobulins consist of a single monoclonal isotype. Type II cryoglobulins consist of monoclonal IgM/polyclonal IgG immune complexes, whereas in type III cryoglobulins, both IgM and IgG are polyclonal (28), and both type II and type III cryoglobulins are mixed cryoglobulins. Compared with patients without cryoglobulinemia, patients with 1 cryoglobulin had less severe hepatic inflammation but worse renal impairment; among those with cryoglobulinemia, patients with 3 cryoglobulins were most likely to have mixed cryoglobulinemia (3, 10, 26) and had the most severe hepatic fibrosis and impaired hepatic synthesis. In particular, no patients with 1 cryoglobulin harbored IgA cryoglobulin, which lessens the possibility of IgA nephropathy, since there is a close relationship between the presence of IgA in cryoglobulinemia immune complexes and its detection in glomeruli (29). All the above results suggested that mixed cryoglobulinemia might develop from the presence of a single cryoglobulin with renal involvement and then progress to the formation of multiple cryoglobulins with frank hepatic involvement, although patients with only one cryoglobulin did not meet the strict criteria for mixed cryoglobulinemia (27). Consistently, the rates of 1 cryoglobulin increased and those of 3 cryoglobulins decreased over time in SVR patients with cryoglobulinemia, supporting the idea that the number of cryoglobulins is correlated with disease stage and progression. Although the positive rates of cryoglobulinemia decreased over time, the cryoglobulins were still detectable in some SVR patients at 4 years posttherapy; some of these patients even had no baseline cryoglobulinemia. The presence of mixed cryoglobulinemia after viral clearance suggests that HCV antigens are not an unfailing component of cryoprecipitating immune complexes (3). Given that CHC massively disturbs the B-cell compartment and generates many large B-cell clones (30), long-term mixed cryoglobulinemia after SVR suggested that some B cells might have reached an HCV‐independent autonomous phase to form pathogenic clones before viral clearance (3). Surprisingly, despite the lack of an immune-modulating effect, DAA seems to have a similar efficacy in eliminating long-term cryoglobulinemia as interferon-based therapy, as the cryoglobulinemia rates decreased from 53.2% in all CHC patients to 15.4% in the SVR patients at 4 years posttherapy in the current study (rate difference: 37.8%) were close to those of patients treated with interferon-based therapy (64.5% in all CHC patients to 22% in SVR patients following interferon-based therapy at 4 posttherapy; rate difference: 42.5%) (3). Moreover, among the 8 patients with baseline cryoglobulinemic vasculitis, 8 (100%) had SVRs, 5 (62.5%) achieved complete clinical response at 12 weeks posttherapy, and 2 of 5 (40%) complete clinical responders experienced relapses between 24 weeks and 3 years posttherapy. Namely, 2 of 8 (25%) SVR patients had cryoglobulinemic vasculitis within 3 years posttherapy. Thus, the efficacy of DAA in treating cryoglobulinemic vasculitis is not superior to that of interferon-based therapy, as the rate for 8-year cryoglobulinemic vasculitis in CHC patients following interferon-based therapy was 23.5% in SVR patients with baseline cryoglobulinemic vasculitis (3). However, we want to stress that although relapses of cryoglobulinemic vasculitis did occur in SVR patients following DAA therapy, the relapse symptoms were milder than baseline symptoms, as the main trigger of cryoglobulinemia, HCV, had been eliminated. This finding was consistent with the notion that most relapses of cryoglobulinemic vasculitis were short‐lived and were less severe than initially experienced before antiviral therapy (11). The precise triggering factors for relapses after SVR were not identified but were reported mainly to be infection and cancer (3) and demand further investigation. Moreover, patients with open wounds, such as severe skin ulcers subsequent to cryoglobulinemic vasculitis (**Figures 2A, B**), were not suitable to receive interferon-based therapy due to the risk of bacterial infection and sepsis subsequent to interferon-related immune modulation (11).

Among SVR patients with baseline cryoglobulinemia, at 12 weeks posttherapy, all signals for cryoglobulinemia had been reversed, as the levels of C3, C4, IgG, IgM and RF (3, 10) all changed in those without 12-week posttherapy cryoglobulinemia, and the reversals persisted for all parameters except for C3 and RF until 48 weeks posttherapy for those without any posttherapy cryoglobulinemia. These findings were consistent with the notions that decreased C4 but not C3 was the unfailing stigma for mixed cryoglobulinemia in untreated CHC patients (31), and RF levels were correlated with the severity of mixed cryoglobulinemia in a dose-dependent manner (3).

Among SVR patients, baseline IgM levels were independently associated with posttherapy cryoglobulinemia until 4 years posttherapy and significantly determined posttherapy cryoglobulinemia in SVR patients with various cut-off values for different posttherapy time points. This was consistent with our previous study based on interferon-based therapy (3) and supported the idea that targeting IgM-positive B cells using rituximab (32) is a feasible strategy for the treatment of HCV-associated cryoglobulinemia, even after achieving an SVR, regardless of anti-HCV regimens used. Interestingly, at 48 weeks and 4 years posttherapy, in addition to baseline IgM, baseline BMI and TC levels were associated with posttherapy cryoglobulinemia, respectively. This finding suggests that the baseline metabolic profile might affect long-term cryoglobulinemia through an unknown mechanism, which demands further investigation.

Overall, during a follow-up of 4 years, the prevalence of cryoglobulinemia decreased from 53.8% in CHC patients to 15.4% in SVR patients following DAA therapy; approximately 1/4 of patients with baseline cryoglobulinemia harbored 1 cryoglobulin; and in patients with cryoglobulinemia, the prevalence rates of 1 cryoglobulin and 3 cryoglobulins increased and decreased over time, respectively. Among patients with baseline cryoglobulinemic vasculitis, 100% had SVRs, and 62.5% experienced complete clinical response at 12 weeks posttherapy; however, 40% of the complete clinical responders experienced relapses 24 weeks to 3 years posttherapy. Baseline IgM levels were crucial for the development of posttherapy cryoglobulinemia in SVR patients. These findings provide important concepts to monitor CHC patients with cryoglobulinemia treated with DAA and pave the way to identifying a target for treating cryoglobulinemia in SVR patients.

## Data Availability Statement

The raw data supporting the conclusions of this article will be made available by the authors, without undue reservation.

## Ethics Statement

The studies involving human participants were reviewed and approved by Chang Chang Memorial Hospital. The patients/participants provided their written informed consent to participate in this study.

## Author Contributions

M-LC, study design and implementation, manuscript drafting, and critical revision of the manuscript for important intellectual content. J-SC, statistics and manuscript writing. Y-HC, L-HP, T-SW, S-CC, and M-YC, data collection and manuscript writing. R-NC, data collection and manuscript writing and critical revision of the manuscript for important intellectual content. All authors contributed to the article and approved the submitted version.

## Funding

This study was supported by grants from the Chang Gung Medical Research Program (CMRPG3I0413,CMRPG3L1191, CMRPG3M0211 and CMRPG1K0111-3) and the National Science Council (MOST 109-2314-B-182-024-, 109-2629-B-182-002-, 110-2629-B-182-001- and 110-2314-B-182-044-) to M-LC. The funders had no role in the study design, data collection and analysis, decision to publish, or preparation of the manuscript. All authors have read the journal’s authorship agreement and policy on disclosure of potential conflicts of interest.

## Conflict of Interest

The authors declare that the research was conducted in the absence of any commercial or financial relationships that could be construed as a potential conflict of interest.

## Publisher’s Note

All claims expressed in this article are solely those of the authors and do not necessarily represent those of their affiliated organizations, or those of the publisher, the editors and the reviewers. Any product that may be evaluated in this article, or claim that may be made by its manufacturer, is not guaranteed or endorsed by the publisher.
