# Supplementary material for: Evolution of Cryoglobulinemia in Direct-Acting Antiviral-Treated Asian Hepatitis C Patients With Sustained Virological Responses: A 4-Year Prospective Cohort Study
Source: Front Immunol. 2022 Mar 8;13:823160. doi: 10.3389/fimmu.2022.823160 (PMC8964347; doi:10.3389/fimmu.2022.823160)
Supplement: Supplementary file 1 [file DataSheet_1.doc]

**Supplementary Table 1. Various DAA combinations used** in the study.

| Genotype 1 |  | Genotype 2 |  | Genotype 3 |  | Genotype 4 |  | Genotype 5 |  | Genotype 6 |  |
| --- | --- | --- | --- | --- | --- | --- | --- | --- | --- | --- | --- |
| DAA | D(W) | DAA | D(W) | DAA | D(W) | DAA | D(W) | DAA | D(W) | DAA | D(W) |
| Asunaprevir (100 mg, bid) with Daclatasvir (60 mg, qd) | 24 | Sovaldi (Sofosbuvir 400 mg qd) with Ribavirin | 12 |  |  |  |  |  |  |  |  |
| Viekirax (Ombitasvir 12.5mg/Paritaprevir 75mg/Ritonavir 50 mg qd) with Exviera (250 mg bid) | 12-24 |  |  |  |  |  |  |  |  |  |  |
| Zepatier (Elbasvir 50 mg/Grazoprevir 100 mg, qd) | 12-16 |  |  |  |  |  |  |  |  |  |  |
| Harvoni (Ledipasvir 90 mg /Sofosbuvir 400 mg qd) with/without Ribavinin (800-1400 mg/day, in two divided doses | 12 | Harvoni (Ledipasvir 90 mg /Sofosbuvir 400 mg qd) with/without Ribavinin (800-1400 mg/day, in two divided doses | 12 |  |  | Harvoni (Ledipasvir 90 mg /Sofosbuvir 400 mg qd) with/without Ribavinin (800-1400 mg/day, in two divided | 12 | Harvoni (Ledipasvir 90 mg /Sofosbuvir 400 mg qd) with/without Ribavinin (800-1400 mg/day, in two divided | 12 | Harvoni (Ledipasvir 90 mg /Sofosbuvir 400 mg qd) with/without Ribavinin (800-1400 mg/day, in two divided | 12 |
| Mavyret (glecaprevir 100mg/pibrentasvir 40mg, 3# qd) | 8-16 | Mavyret (glecaprevir 100mg/pibrentasvir 40mg, 3# qd) | 8-12 | Mavyret (glecaprevir 100mg/pibrentasvir 40mg, 3# qd) | 8-16 | Mavyret (glecaprevir 100mg/pibrentasvir 40mg, 3# qd) | 8-12 | Mavyret (glecaprevir 100mg/pibrentasvir 40mg, 3# qd) | 8-12 | Mavyret (glecaprevir 100mg/pibrentasvir 40mg, 3# qd) | 8-12 |
| Epclusa (sofosbuvir 400 mg/velpatasvir 100 mg qd) with or without Ribavirin | 12 | Epclusa (sofosbuvir 400 mg/velpatasvir 100 mg qd) with or without Ribavirin | 12 | Epclusa (sofosbuvir 400 mg/velpatasvir 100 mg qd) with or without Ribavirin | 12 | Epclusa (sofosbuvir 400 mg/velpatasvir 100 mg qd) with or without Ribavirin | 12 | Epclusa (sofosbuvir 400 mg/velpatasvir 100 mg qd) with or without Ribavirin | 12 | Epclusa (sofosbuvir 400 mg/velpatasvir 100 mg qd) with or without Ribavirin | 12 |

DAA: direct-acting antiviral agent; D: duration: W: weeks.

**Supplementary Table 2. Comparisons between CHC patients with and without cryoglobulinemia.**

|  | Cryoglobulinemia (-) (n=195, C 0) | 1 cryoglobulins  (n=54, C 1) | 3 Cryoglobulins  (n=116, C 3) | *p* values  C 0 vs. C 1 | *p* values  C 0 vs C 3 |
| --- | --- | --- | --- | --- | --- |
| Female, n (%) | 93 (47.7) | 29 (53.7) | 71 (61.2) | 0.145 | 0.048 |
| Age (years) | 59.1+/-12.9 | 59.8+/-12.85 | 62.9+/-12.7 | 0.924 | 0.003 |
| BMI (kg/m^2^) | 24.9+/-3.9 | 23.44+/-3.37 | 24.4+/-3.87 | 0.002 | 0.398 |
| Log HCV RNA (logIU/mL) | 6.14+/-0.83 | 6.03+/-0.85 | 5.76+/-0.81 | 0.412 | <0.001 |
| HCV genotype |  |  |  |  |  |
| Genotype 1, n (%) | 118 (60.5) | 30 (55.6) | 70 (60.3) | 0.507 | 0.963 |
| Genotype 2, n (%) | 55 (28.1) | 18 (33.3) | 33 (28.4) | 0.502 | 0.893 |
| Genotype 3, n (%) | 1 (0.5) | 1 (1.9) | 4 (3.4) | 0.338 | 0.104 |
| Others, n (%) | 21 (10.7) | 5 (9.2) | 9 (7.7) | 0.678 | 0.278 |
| ALT(U/L) | 83.9+/-118.6 | 53.8+/-39.2 | 88.5+/-60.7 | 0.001 | 0.287 |
| FIB-4 | 2.65+/-2.29 | 2.74 +/-2.41 | 5.56+/-4.42 | 0.988 | <0.001 |
| Platelet (10^3^/mm ) | 190.6+/-68.8 | 191.9+/-73.9 | 143.6+/-68.1 | 0.559 | <0.001 |
| Liver cirrhosis, n (%) | 25 (16.7) | 8 (14.8) | 40 (34.5) | 0.702 | <0.001 |
| eGFR (ml/min/1.73m^2^) | 91.5+/-40.5 | 75.7+/-43.7 | 84.3+/-40.9 | 0.01 | 0.097 |
| Total cholesterol (mg/dL) | 176.1+/-33.6 | 168.2+/-41.0 | 162.0+/-36.0 | 0.665 | <0.001 |
| Triglycerides (mg/dL) | 103.5+/-46.3 | 116.9+/-70.6 | 92.2+/-38.7 | 0.144 | 0.033 |
| HOMA-IR | 3.34+/-6.30 | 2.67+/-3.74 | 3.06+/-3.01 | 0.383 | 0.805 |
| C3 (mg/dL) | 104.4+/-20.2 | 97.8+/-15.5 | 93.0+/-18.6 | 0.251 | <0.001 |
| C4 (mg/dL) | 21.3+/-7.4 | 18.9+/-7.5 | 16.5+/-7.3 | 0.254 | <0.001 |
| IgG (mg/dL) | 1675+/-442 | 1696.5+/-388 | 2017+/-500 | 0.825 | <0.001 |
| IgM (mg/dL) | 90.5+/-42.4 | 134.0+/-62.2 | 145.1+/-70.4 | <0.001 | <0.001 |
| RF(IU/mL) | 15.2+/-20.6 | 14.03+/-7.45 | 30.6+/-75.9 | 0.656 | 0.016 |

CHC: chronic hepatitis C virus infection; C 0: cryoglobulinemia negative; C 1: 1 cryoglobuline; C 3: 3 cryoglobulinemins; BMI: body mass index; IgG: Immunoglobulin G; IgA: Immunoglobulin A; IgM: Immunoglobulin M; ALT: alanine transaminase; FIB-4: Fibrosis-4; eGFR: estimated glomerular filtration rate;; HOMA-IR: homeostasis model assessment-insulin resistance; C3: complement component 3; C4: complement component 4; IgG: Immunoglobulin G; IgM: Immunoglobulin M; RF: rheumatoid factor.

**Table 3. Longitudinal evolution of cryoglobulinemia of 353 SVR patients**

| Baseline C | 12W PT  C (+), n (%) | 24W PT  C (+), n (%) | 48W PT  C (+), n (%) | 72W PT  C (+), n (%) | 96W PT  C (+), n (%) | 120W PT  C (+), n (%) | 144W PT  C (+), n (%) | 168W PT  C (+), n (%) | 192W PT C(+), n (%) |
| --- | --- | --- | --- | --- | --- | --- | --- | --- | --- |
| Negative (n=169) | 28 (16.56) | 27 (15.9) | 13 (7.9) | 16 (11.5) | 10 (10.7) | 10 (11.1) | 5 (8.47) | 4 (9.3) | 3 (9.0) |
| Positive (n=184) | 77 (41.8) | 56 (30.4) | 51 (27.7) | 35 (23.8) | 26 (24.3) | 26 (26.8) | 20 (27.3) | 16 (27.5) | 13 (27.6) |

SVR: Sustained virological response. W: week; PT: post-therapy; C: cryoglobulinemia; N: number

**Supplementary Table 4. Comparisons between baseline and post-therapy variables in CHC patients with cryoglobulinemia and SVR patients without post-therapy cryoglobulinemia.**

|  | Baseline vs. 12 weeks post-therapy | | Baseline vs. 24 weeks post-therapy | | Baseline vs. 48 weeks post-therapy | |
| --- | --- | --- | --- | --- | --- | --- |
|  | Post-Pre therapy  difference | *p* values | Post-Pre therapy  difference | *p* values | Post-Pre therapy  difference | *p* values |
| C3 (mg/dL) | 4.98 ± 13.2 | 0.002 | 6.23+/-13.78 | 0.01 | 4.96 ± 15.0 | 0.351 |
| C4 (mg/dL) | 1.67 ± 4.55 | 0.003 | 2.03 ± 3.78 | <0.001 | 3.10 ± 3.31 | 0.023 |
| IgG (mg/dL) | -388.1 ± 306.1 | <0.001 | - 503.5 ± 313.5 | <0.001 | - 1040 ± 344.2 | <0.001 |
| IgM (mg/dL) | -25.7 ± 23.1 | <0.001 | -38.2 ± 28.0 | <0.001 | -36.9 ± 21.5 | <0.001 |
| RF (IU/mL) | -2.34 ± 7.55 | 0.01 | -11.6 ± 67.3 | 0.173 | -4.75 ± 25.1 | 0.115 |

C3: complement component 3; C4: complement component 4; IgG: Immunoglobulin G; IgM: Immunoglobulin M; RF: rheumatoid factor.

**Supplementary Table 5. Univariate and multivariate analyses of factors for 12-week post-therapy cryoglobulinemia in SVR patients.**

|  | **Univariate** | | **Multivariate** | |
| --- | --- | --- | --- | --- |
|  | OR (95% CI OR) | *p* values | OR (95% CI OR) | *p* values |
| Male, yes | 0.64 (0.398-1.027 ) | 0.064 | 0.648 (0.336-1.250) | 0.165 |
| Age (years) | 1.015 (0.996-1.034) | 0.119 |  |  |
| BMI (kg/m^2^) | 0.984 (0.928-1.044) | 0.592 |  |  |
| ALT(U/L) | 0.999 (0.996-1.002) | 0.504 |  |  |
| FIB-4 | 1.098 (1.029-1.172) | 0.005 | 1.045 (0.918-1.191) | 0.505 |
| Platelet (10^3^/mm ) | 0.995 (0.992-0.999) | 0.005 | 1.000 (0.992-1.008) | 0.937 |
| Liver cirrhosis, n (%) | 1.575 (0.918-2.702) | 0.099 | 1.274 (0.571-2.844) | 0.554 |
| eGFR (ml/min/1.73m^2^) | 0.995 (0.989-1.001) | 0.083 | 0.993 (0.984-1.001) | 0.127 |
| Total cholesterol (mg/dL) | 0.988 (0.981-0.996) | 0.002 | 0.999 (0.988-1.011) | 0.93 |
| Triglycerides (mg/dL) | 0.996 (0.991-1.001 ) | 0.135 |  |  |
| HOMA-IR | 0.944 (0.859-1.038) | 0.237 |  |  |
| C3 (mg/dL) | 0.988 (0.975-1.001) | 0.073 | 0.988 (0.971-1.007) | 0.095 |
| C4 (mg/dL) | 0.965 (0.932-0.999) | 0.045 | 1.031 (0.987-1.078) | 0.173 |
| IgG (mg/dL) | 1.001 (1.000-1.001) | 0.008 | 1.000 (1.000-1.001) | 0.211 |
| IgM (mg/dL) | 1.014 (1.009-1.020) | <0.001 | 1.009 (1.004-1.014) | 0.001 |
| RF(IU/mL) | 1.005 (0.998-1.013) | 0.166 |  |  |

SVR: sustained virological response; OR: Odds ratio; CI: confidence invertal; BMI: body mass index; ALT: alanine transaminase; FIB-4: Fibrosis-4; eGFR: estimated glomerular filtration rate; HOMA-IR: homeostasis model assessment-insulin resistance; C3: complement component 3; C4: complement component 4; IgG: Immunoglobulin G; IgM: Immunoglobulin M; RF: rheumatoid factor.

**Supplementary Table 6. Univariate and multivariate analyses of factors for 24-week post-therapy cryoglobulinemia in SVR patients.**

|  | **Univariate** | | **Multivariate** | |
| --- | --- | --- | --- | --- |
|  | OR (95% CI OR) | *p* values | OR (95% CI OR) | *p* values |
| Male, yes | 0.563 (0.322 -0.985) | 0.044 | 0.563 (0.322 -0.985) | 0.196 |
| Age (years) | 1.022 (1.000-1.045) | 0.053 | 1.022 (1.000-1.045) | 0.053 |
| BMI (kg/m^2^) | 0.986 (0.921-1.056) | 0.69 |  |  |
| ALT(U/L) | 0.999 (0.996-1.003) | 0.62 |  |  |
| FIB-4 | 1.130 (1.050-1.215) | 0.001 | 1.130 (1.050-1.215) | 0.001 |
| Platelet (10^3^/mm ) | 0.994 (0.990-0.998) | 0.007 | 0.994 (0.990-0.998) | 0.007 |
| Liver cirrhosis, n (%) | 2.171 (1.192-3.954) | 0.011 | 2.171 (1.192-3.954) | 0.011 |
| eGFR (ml/min/1.73m^2^) | 0.994 (0.987-1.001) | 0.086 | 0.994 (0.987-1.001) | 0.086 |
| Total cholesterol (mg/dL) | 0.997 (0.989-1.005 ) | 0.423 |  |  |
| Triglycerides (mg/dL) | 0.994 (0.987-1.000 ) | 0.066 | 0.994 (0.987-1.000 ) | 0.066 |
| HOMA-IR | 0.970 (0.892-1.055) | 0.478 |  |  |
| C3 (mg/dL) | 0.979 (0.964-0.994) | 0.007 | 0.979 (0.964-0.994) | 0.007 |
| C4 (mg/dL) | 0.973 (0.921-1.026) | 0.167 |  |  |
| IgG (mg/dL) | 1.001 (1.000-1.001) | 0.008 | 1.001 (1.000-1.001) | 0.008 |
| IgM (mg/dL) | 1.011 (1.006-1.016) | <0.001 | 1.011 (1.006-1.016) | <0.001 |
| RF(IU/mL) | 0.998 (0.990-1.006) | 0.653 |  |  |

SVR: sustained virological response; OR: Odds ratio; CI: confidence invertal; BMI: body mass index; ALT: alanine transaminase; FIB-4: Fibrosis-4; eGFR: estimated glomerular filtration rate; HOMA-IR: homeostasis model assessment-insulin resistance; C3: complement component 3; C4: complement component 4; IgG: Immunoglobulin G; IgM: Immunoglobulin M; RF: rheumatoid factor.
